# Integration of UAV Photogrammetry and SPH Modelling of Fluids to Study Runoff on Real Terrains

**DOI:** 10.1371/journal.pone.0111031

**Published:** 2014-11-05

**Authors:** Anxo Barreiro, Jose M. Domínguez, Alejandro J. C. Crespo, Higinio González-Jorge, David Roca, Moncho Gómez-Gesteira

**Affiliations:** 1 EPHYSLAB Environmental Physics Laboratory, Universidade de Vigo, Ourense, Spain; 2 Applied Geotechnology Group. Department of Natural Resources & Environmental Engineering, Universidade de Vigo, Vigo, Spain; University of Missouri, United States of America

## Abstract

Roads can experience runoff problems due to the intense rain discharge associated to severe storms. Two advanced tools are combined to analyse the interaction of complex water flows with real terrains. UAV (Unmanned Aerial Vehicle) photogrammetry is employed to obtain accurate topographic information on small areas, typically on the order of a few hectares. The Smoothed Particle Hydrodynamics (SPH) technique is applied by means of the DualSPHysics model to compute the trajectory of the water flow during extreme rain events. The use of engineering solutions to palliate flood events is also analysed. The study case simulates how the collected water can flow into a close road and how precautionary measures can be effective to drain water under extreme conditions. The amount of water arriving at the road is calculated under different protection scenarios and the efficiency of a ditch is observed to decrease when sedimentation reduces its depth.

## Introduction

Surface runoff is the amount of water that cannot infiltrate soils and drains. This excess water can come from rain or snowmelt and can flow over saturated soils and any impervious surfaces such as roofs, roads, and sidewalks.

The passage of strong storms through the Northwest corner of the Iberian Peninsula is frequently accompanied by heavy showers that result in high rainfalls during short periods of time, typically on the order of 10–20 minutes. Although these areas have historically suffered the passage of continuous fronts, the weather conditions have worsened during the last decades and the area is now more prone to extreme rain events than it was in the past. Given these increasingly heavy rains can cause problems in the stability of road slopes, walls, and bridge abutments, road designs must be updated. Therefore, it is necessary to establish a framework to mitigate the effects of strong runoffs both on population and on the economic activity of the areas at risk to such events.

Two advanced tools are combined in this work. On the one hand, UAV (Unmanned Aerial Vehicle) photogrammetry is employed to obtain accurate topographic information of the zone. On the other hand, the so called SPH (Smoothed Particle Hydrodynamics) technique allows modelling complex flows on uneven geometries. UAV systems are been successfully applied to the field of geomatics over the last few years. UAV platforms have become a new tool for remote sensing that can operate under circumstances where the traditional airborne platforms cannot, due mainly to cost, lack of flexibility or danger issues. When UAVs operate in combination with digital cameras, they become photogrammetric measurement platforms that can provide useful topographic data [1]. UAV photogrammetry represents a low-cost alternative to the classical manned aerial photogrammetry. In addition, UAVs can work at lower altitude than manned systems and it is possible to achieve very high resolution and accuracy with midrange photographic equipment [2].

Traditional computational fluid dynamics (CFD) techniques such as volume-of-fluid methods (VOF) have been used in the study of free-surface problems. Nevertheless, the Eulerian numerical methods such as those based on finite volume technique require expensive mesh generation and present severe technical challenges associated with implementing conservative multi-phase schemes that can capture the nonlinearities within rapidly changing geometries. The emergence of meshless schemes has provided a much needed alternative. Developed originally for astrophysics in the 70′s, the meshless method Smoothed Particle Hydrodynamics (SPH) has been developed rapidly during the last decade for its application in engineering. The method uses particles to represent a fluid and these particles move according to the governing dynamics. When simulating free-surface flows, the Lagrangian nature of SPH allows the domain to be multiply-connected, with no need of a special treatment of the surface, making the technique ideal for studying violent free-surface motion. Each particle represents a point where physical quantities are computed as an interpolation of the values of the nearest particles. Thereby, SPH computes the trajectories of the fluid particles, which interact according to the Navier-Stokes equations, and computes density, pressure, and velocity of the particles, so that, the flow is reproduced and its interaction with complex geometries can be numerically studied. One of the main advantages of the Lagrangian approach used in SPH methods relies on the fact that meshless models do not need a specific wetting-drying treatment, which is a key point in Eulerian models. These kind of situations, where a part of the terrain passes from dry to wet (or vice-versa), appear in models for river inundation, dam-breaks or tsunami simulations where run-up should be determined with accuracy. For a complete review on wetting-drying models the reader is referred to [3].

The DualSPHysics ([4], [5], [6]) code has been developed to apply the SPH technique to real engineering problems. The software can be run both on CPUs and GPUs (graphics cards with powerful parallel computing), is open source and can be freely downloaded from Dual SPH Physics [7]. Details on the implementation of DualSPHysics can be found in [8], [9], [10]. The model has been successfully applied to the study of some engineering problems such as the computation of forces exerted by large waves on the urban furniture [11] or the study of the run-up in an armour block breakwater [12].

In the present work, DualSPHysics is applied to a real case to simulate extreme runoffs on a terrain whose geometry was obtained using UAV photogrammetry. The study case simulates how the collected water can flow into a close road and the countermeasures to decrease these kind of pernicious events. Intense rain events are simulated using a water inflow that mimics extreme meteorological conditions in the area of study. The amount of water arriving at the road is calculated under different protection scenarios. The effect of a ditch to prevent water flow onto the road is analysed. The efficiency of this precautionary measure is observed to decrease when sedimentation reduces its depth.

## Methods

### UAV technology

The present work requires a 3D model of the study area as the basis for the hydrodynamic evaluation using DualSPHysics. The site area is relatively small (around 6 ha) and the required resolution is high (about 5 cm) to detail water paths. For all these factors, the use of a combined technique of photogrammetry and UAV system seems a good choice.

The aerial inspection unit used for this work was an eight propeller UAV, the Okto XL from Mikrokopter [13]. This low-cost UAV consists of a frame of aluminium square tubes and carbon fibre base plates. The system is powered by eight MK-3638 brushless motors and APC SlowFly 12×3.8 propellers. These motors are controlled by a Brushless Control V2.0 that sets the rotary speed of each of them separately.

The UAV is controlled by the Flight Control, which includes a three axis accelerometer, a three axis gyroscope, and a pressure sensor used to calculate the relative height and orientation around X and Y axis (roll and pitch angles, respectively), and improve the stability of the UAV. A Navi-Control board with a magnetic compass and an ublox LEA-6 H GPS module is used to send position (geo-reference) and orientation around Z axis (heading angle) data to the Flight Control module during flight in order to complement the information provided by its inner sensors. Sony Nex 7 camera has been mounted on the aerial unit [14]. This camera allows the capture of RGB images (RAW and JPG formats) with a resolution of 24.3 MP (approximately 6000×4000 active pixels; 3∶2). It mounts a CMOS sensor (Exmor APS-C HD) with 23.5 mm×15.6 mm size, ISO 100–16000, and 2.4 m dot OLED Electronic View Finder (EVF). Shutter speed is 1/4000–30 and allows up to 10 frames per second. The dimensions of the camera are 119.9×66.9×42.6 mm, with a weight of 353 g. Mounted lens is a Sony SEL16F28 (focal length of 16 mm and F2.8). The viewing angle of the lens is 83°. Diameter of the lens is 62 mm, filter size 49 mm, length 22.5 mm, and weight 70 g. The camera mount is the MK HiSight SLR1, also from Mikrokopter [13]. It uses two servomotors for the compensation of the roll and pitch angles and for maintaining the sensor stabilized. A Graupner Hott MX-20 transmitter has been used to establish the initial orientation of the camera mount and to remotely pilot the UAV. This remote control transmits commands to the Graupner GR-24 receiver, located on the UAV and connected to the Flight Control. Flight high is situated between 30 m and 50 m above ground. The flight is always performed on the same horizontal plane and the change in the flight high comes from the differences of the terrain. Taking into account the resolution in pixels of the sensor, the viewing angle of the lens, and the flight high, the obtained spatial resolution achieves values from 1.38 pixel/cm (30 m height) to 0.75 pixel cm (50 m height).

**Figure 1:**
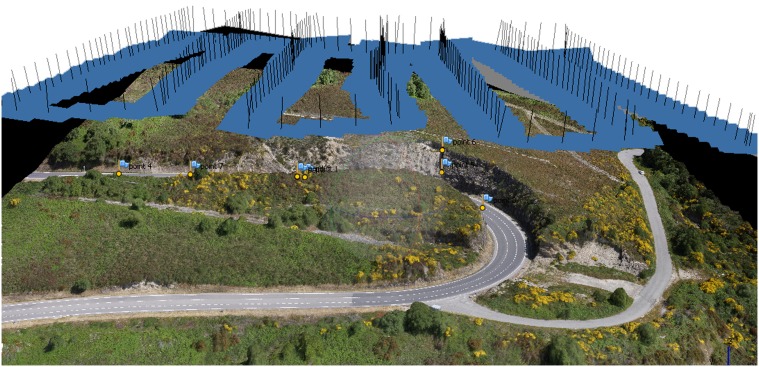
3D model obtained with Photoscan software from UAV imagery. The camera position during the UAV survey is shown using blue planes. The normal vector of the plane is presented with a black line.

Data acquisition was first planned in the laboratory using Google Earth geo-referenced images and the Mikrokopter planning software to generate the GPS waypoints for the survey. This way points define the trajectory of the UAV and the place where an image is taken (Figure 1). Once the geographic coordinates were calculated, they were uploaded to the UAV firmware. The ground – UAV height and the distance between waypoints was chosen in a way that the longitudinal and transversal images overlap approximately 60%. This operation is essential for proper post-processing photogrammetric restitution. The pilot was responsible for the take-off and landing operations using the remote control from ground. The waypoint positioning and image acquisition was done autonomously by the UAV. Photoscan is the software-suite used for photogrammetric restitution. Photoscan photogrammetric software uses computer vision algorithms, based on grey level matching, to the automated extraction of common points on UAV photographs and performs the photogrammetric restitution [15]. The common points allow obtaining the camera calibration and relative orientation of the images in an arbitrary coordinate system using a fundamental matrix [16], [17]. Once the angular and spatial relative position of the images has been obtained, bundle adjustment algorithm is carried out through iterative and least square process based on the colinearity condition to refine the 3D coordinate calculation. The absolute orientation is completed with six topographic points (terrain coordinates) taken with a total station from the road under study and its neighbourhood. At the end of the process a geo-referenced 3D point cloud with the geometric information of the area is obtained (Figure 1).

### Smoothed Particle Hydrodynamics model

We will only refer here to the main issues of the SPH theory and the governing equations implemented in the DualSPHysics code. A complete review of the SPH formulation, in general, and the DualSPHysics implementation, in particular, can be seen in [5], [6], [18], [19].

As mentioned in the Introduction section, Smoothed Particle Hydrodynamics is a Lagrangian and meshless method where the fluid is discretised into a set of particles. Each particle is the point where physical quantities (such as position, velocity, density, pressure) are computed as an interpolation of the values of the neighbouring particles. The contribution of the nearest particles is weighted according to distance between particles and a kernel function (*W*) is used to measure this contribution depending on the inter-particle distance that is defining using a smoothing length (*h*). The smoothing length is a characteristic length used to define the area of influence of the kernel and the kernel presents compact support to not consider contributions with other particles beyond the smoothing length.

The mathematical fundamental of SPH is based on integral interpolants, therefore any function can be computed by the integral approximation:
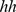


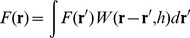
(1)


The kernel functions must fulfil several properties ([20]), such as positivity inside the area of interaction, compact support, normalization and monotonically decreasing with distance. One option is the quintic kernel by [21] where the weighting function vanishes for inter-particle distances greater than 2 *h* and defined in 3D as:
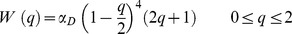
(2)where *q = r/h* and *α_D_* = 21/(16πh^3^) is the normalization constant. Following [22], results show that the best compromise between accuracy and time computation cost is reached by the use of the Wendland kernel. In general, the higher the order of the kernels, the greater the accuracy of the SPH scheme.

The function *F* in Eq. (1) can be expressed in a discrete form based on the particles. Thus, the approximation of the function is interpolated at particle *a* and the summation is performed over all the particles within the region of compact support of the kernel:
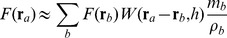
(3)where the volume associated to the neighbouring particle *b* is *m_b_*/*ρ_b_*, with m and *ρ* being the mass and the density, respectively.

In the classical SPH formulation, the Navier-Stokes equations are solved and the fluid is treated as weakly compressible (e.g. see [19]). The conservation laws of continuum fluid dynamics, in the form of differential equations, are transformed into their particle forms by the use of the kernel functions.

The momentum equation proposed by [20] has been used to determine the acceleration of a particle (*a*) as the result of the particle interaction with its neighbours (particles *b*):
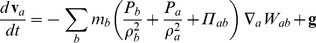
(4)being v velocity, *P* pressure, *ρ* density, m mass, **g** = (0,0,–9.81) m·s^−2^ the gravitational acceleration and *W_ab_* the kernel function that depends on the distance between particle a and b. *Π_ab_* is the viscous term according to the artificial viscosity proposed in [20]. This artificial viscosity can be calculated as
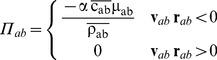
(5)with 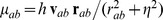, 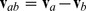, 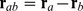 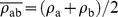, *η* ^2^ = 0.01 h^2^ and α is a coefficient that needs to be tuned in order to introduce the proper dissipation.

Artificial viscosity is by far the most used approach to describe viscosity in SPH. The method, which is purely phenomenological, was firstly proposed by Monaghan ([20]) and depends (for subsonic flows) on only one free parameter (*α*) that is externally tuned. More sophisticated approaches can be seen in [23]. In particular, the so called Sub-Particle Scale (SPS) approach is also available in the DualSPHysiscs package and it has been used in different studies ([24], [25]). Nevertheless, [19] compared experimental data with numerical results obtained for different approaches, showing comparable accuracy. Thus, the artificial viscosity approach was adopted in the present study since, on the one hand, it provides a satisfactory level of accuracy and, on the other hand, the interaction fluid-fluid and fluid-terrain can be computed easily tuning the alpha parameter.

The parameter *α*, which is externally fixed, depends both on the initial distance among particles and on the smoothing length (*h*). Here, two different values of *α* were considered to mimic the interaction among fluid particles (*α_FF_*) and among fluid and boundary particles (*α_FB_*). This last parameter is critical due to the different nature of the terrain. In laboratory experiments, where the bottom is made of Plexiglas and exerts little influence on water propagation, *α_FF_* = *α_FB_* is usually adopted ([19]). Nevertheless, in real cases the roughness of terrain plays a key role on water propagation. The procedure followed to obtain both *α* values will be described later.

The mass of each particle is constant, so that changes in fluid density are computed by solving the conservation of mass or continuity equation in SPH form:
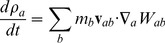
(6)


In this approach, the fluid is treated as weakly compressible [26], which facilitates the use of an equation of state to determine fluid pressure, which is much faster than solving an equation such as the Poissons equation. Hence, following the Tait’s equation of state ([27]), the relationship between pressure and density is assumed to follow the expression:
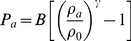
(7)where γ is the polytrophic constant equal to 7, *ρ_0_* is the reference density of the fluid, 1000 kg m^−3^ in the case of water, and 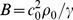 sets a limit for the maximum change in the density.

This compressible fluid permits a speed of sound, *c*, which is given by square root of the derivative of this equation of state with respect to density and where *c_0_* is the speed of sound a the reference density (at the surface of the fluid):
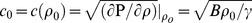
(8)


Therefore, the choice of B is going to play a key role since it determines the speed of sound. The compressibility is adjusted to slow the speed of sound so that the time step in the model (using a Courant condition based the speed of sound) is reasonable. Another limitation on compressibility is to restrict the sound speed to be at least ten times faster than the maximum fluid velocity, thereby keeping density variations to within less than 1% as suggested by [26].

In the SPH model used in this work, boundaries are represented as a set of boundary particles that exert a repulsive force on the fluid particles when they approach. The dynamic boundary conditions described in [28] are used. Following this approach, the boundary particles satisfy the same continuity equation as the fluid particles, therefore, their density and pressure can also evolve. In this way, when a fluid particle approaches a boundary particle, and they are at the interaction defined by the kernel range, the density of the boundary particles increases giving rise to a pressure increase in in such a way that the force exerted on the fluid particle also increases due to the pressure term in the momentum equation, which creates a repulsive mechanism between fluid and boundary. Note that even if the momentum equation is carried out for the interaction between fluid and boundary particle, the force exerted onto the boundary is not used to compute its movement since the boundaries will remain fixed or will move according to some external pre-imposed movement.

### Data Pre-Processing

The photogrammetric data acquisition leads to a point cloud with accurate geometric information of the scene. It is necessary to convert that set of points into SPH particles, in particular, the geometry of a scenario will be converted into boundary SPH particles and the flow will be simulated using fluid SPH particles. The conversion of the point cloud into SPH particles is carried out by using surface geometries (described by triangles) as an intermediate step. Thereby, a conversion *POINTS to TRIANGLES* is firstly performed and *TRIANGLES to SPH particles* is finally carried out.

### POINTS to TRIANGLES

**Figure 2:**
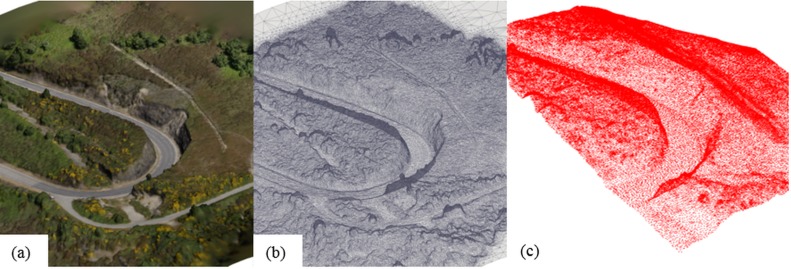
Digital Surface Model (DSM) of the test site in STL format (colourised (a) and no colourised (b)) and then converted into SPH particles (c).

The point cloud generated by Photoscan is triangulated to obtain a polygon mesh using 2D Delaunay triangulation. A mesh is a Delaunay triangulation if all circumcircles of all triangles of the network are empty. The mesh is colourised using the radiometric information from the images and the orientation of the camera. The colours can be useful to identify the type of terrain, materials, etc. The generation of the mesh is crucial because it closes holes on the geometry and allows the realistic water displacement without abnormal infiltration. Therefore, a STL file can be produced as seen in Figure 2a (colourised) and Figure 2b (no colourised). This file format describes a raw triangulated surface by the unit normal and vertices of the triangles using a 3D Cartesian coordinate system.

### TRIANGLES to SPH particles

Starting from those triangles (unit normal and vertices), SPH particles are created to reproduce the boundary layer of the realistic geometry. The pre-processing tool employed in DualSPHysics is a drawing application that creates points that will be converted into particles which carry physical quantities (position, velocity, density…). It creates the configuration that will be loaded by the SPH solver as initial condition for the simulation. The central feature of the code is its capability to convert a wide variety of geometrical shapes into their respective particle representation. In fact it is possible to convert any shape that consists of a mesh with edges and faces. The procedure is based on a simple algorithm. It employs a 3D mesh to locate points which represent possible particle positions. The main idea is to build an object by placing particles only at those points which are required to generate the desired geometry, so that the position of the particles is defined by the 3D mesh and depends on its resolution.

An initial distance *dp* is defined to create the 3D mesh, so that the nodes of the mesh are equidistant. Firstly, the mesh nodes around the object are defined and then particles are created only in the nodes that compose the shape of the desired geometry. For the case of triangles, only the nodes at a distance less than 0.6*dp to one of the sides of the triangle are used to create particles. In this way, the accuracy of the spatial resolution depends on the mesh resolution. In addition, complex 3D models such as STL files can be imported, split into different triangles and each triangle can be converted into particles as described (Figure 2c). More detailed information about the pre-processing can be found in [29].

### Case of Study

The geometries shown in Figures 1 and 2 correspond to the scenario of the case of study. More specifically, the road section selected for this study (see Figure 2a) is located around 42.2946°N and 7.5888°W. The rainfall causes important damages and significant geomorphic changes that are visible in the surrounding orography. These kind of incidents motivates the current work and justifies the test site selection. The slopes of the road are mainly formed by shale that is a rock of natural water proofing, consequently, infiltration is minimal and high road runoffs are frequently generated during rainfall events in this stretch. Height oscillates between 10 m and 15 m. Here we will focus on the sloped terrain above the road side walls where water flows downhill under during rain events.

**Figure 3:**
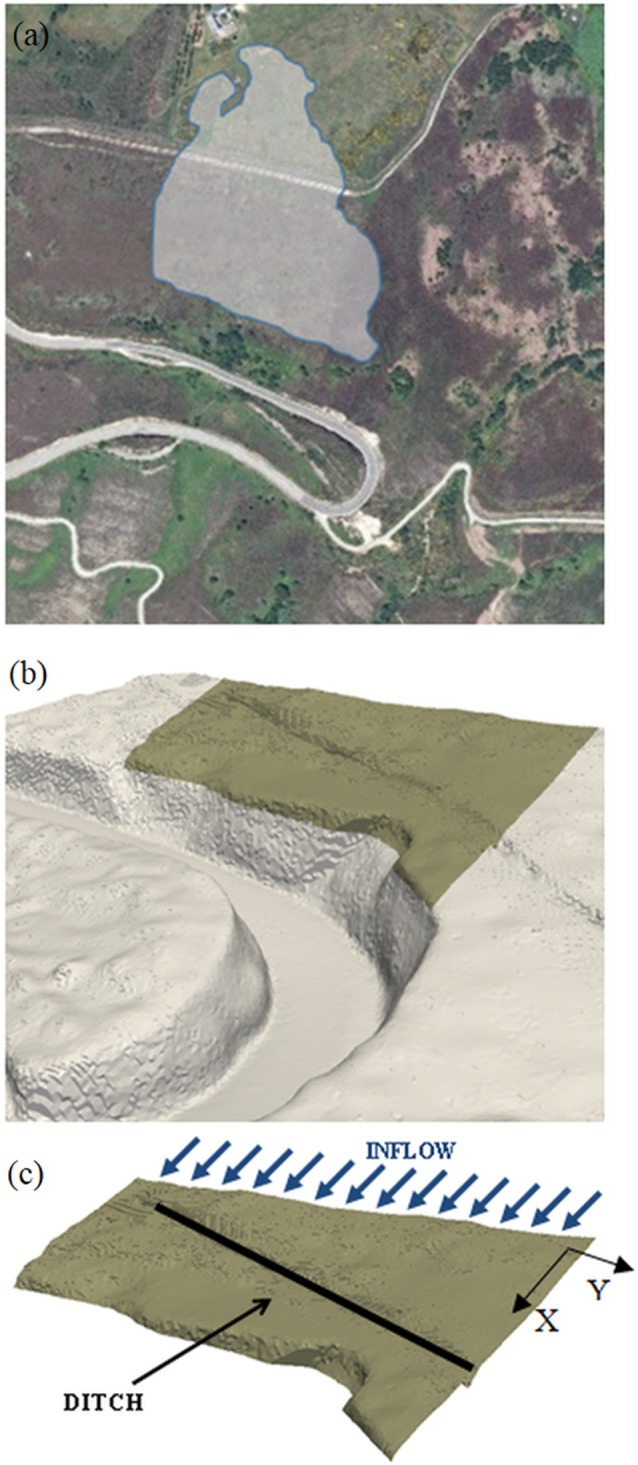
Domain of the SPH simulation and location of the ditch: watershed (a) that concentrates water on the region of interest (b) and the upper boundary where the inflow is imposed (c).

Figure 3 shows the domain of the numerical simulation using DualSPHysics code. The watershed (shaded area in Fig. 3a) is the catchment area that concentrates water on the region of interest (Fig. 3b). Thus, the watershed outlet corresponds to the upper boundary of the region under study where the inflow is imposed as shown in Fig. 3c. Note that the dynamic boundary conditions described above are only used to simulate the ground. Inlet/outlet conditions were considered for the rest of the boundaries. The lower and lateral boundaries are open, in such a way that particles can go through them and leave the domain. Inflow conditions were considered for the upper boundary. These conditions allow fixing the instantaneous number of particles that enter the computational domain in such a way that the water inflow can be controlled. The darker surface in Fig. 3b, which has been magnified in Fig. 3c corresponds to the SPH numerical area, which is 83 m×75 m with a steep slope (∼17%) in X direction and a gentler slope (∼7%) in Y direction. Particles are initially located with an inter-particle spacing of 0.04 m. The presence of a ditch across the terrain can also be observed in the figure. That ditch, which is approximately 0.80 m deep and 0.5 m wide, was built as a preliminary precautionary measure to prevent flooding on the road. Different simulations, with and without the ditch, have been designed.

### Numerical setup

Table 1 summarises the main SPH parameters used in DualSPHysics to perform the simulations of this work.

**Table 1:** SPH parameters in DualSPHysics.

Initial interparticle distance	*dp* = 0.04 m
Smoothing length	*h* = 0.06 m
Time integrator	Predictor-corrector algorithm
Kernel Function	Wendland kernel ([21])
Viscosity Treatment	Artificial viscosity ([20]) *α_FF_* = 0.2
Equation of State	Tait equation ([27])
Boundary Condition	Dynamic BCs ([28])

### Inflow conditions

**Figure 4:**
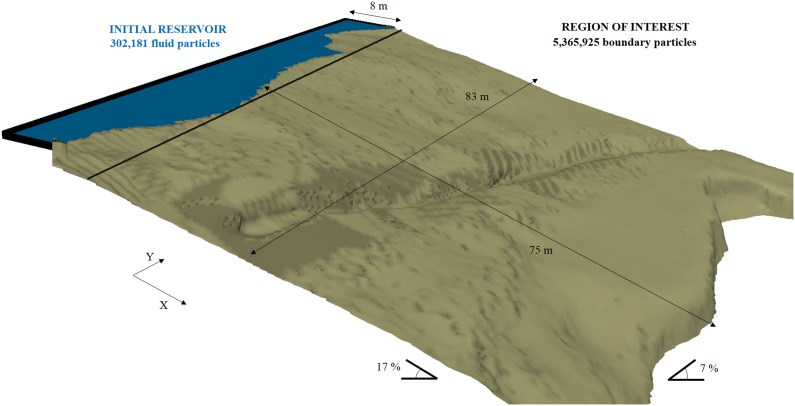
Domain of the SPH simulation and reservoir to create the desired inflow rate.

A maximum daily precipitation of *P* = 96 mm day^−1^ was estimated for the area under study according to data provided by the Spanish Meteorological Agency (AEMET) considering a return period of 50 years [30]. A concentration time of approximately 0.1 hour was calculated by using the length (L = 200 m), the slope (S = 0.17) and the area (A = 0.045 km^2^) of the watershed following the expression 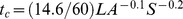, according to [31]. The discharge (in m^3^s^−1^) was calculated following 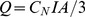, where *C_N_* = 0.3 is the runoff curve number obtained from [32], *A* is the watershed area and *I_t_* is the intensity of the precipitation that can be calculated for the concentration time according to 
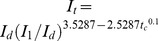
 following [32], where *I_d_*  = *P*/24 is the intensity per hour and *I_1_* = 9 *I_d_*, according to tabulated data for the area provided by [32]. Using this rough approximation, the obtained discharge is on the order of 0.5 m^3^s^−1^. Therefore, a discharge similar to the previous one is desired to be simulated using DualSPHysics code. Special inlet conditions were implemented for these simulations. New fluid particles are being inserted at the upper boundary as shown in Fig. 3c (blue arrows). A numerical reservoir is created beyond the upper part of the region of interest (Figure 4). The reservoir is initially filled with fluid particles and these ones only enter the domain when overflow. Layers of particles are created above the existing ones in the reservoir at a rate that also determines the overflow. The numerical inflow needs to be 0.5 m^3^s^−1^, each m^3^ contains 17,802 particles so 8,901 particles are being inserted per second.

**Figure 5:**
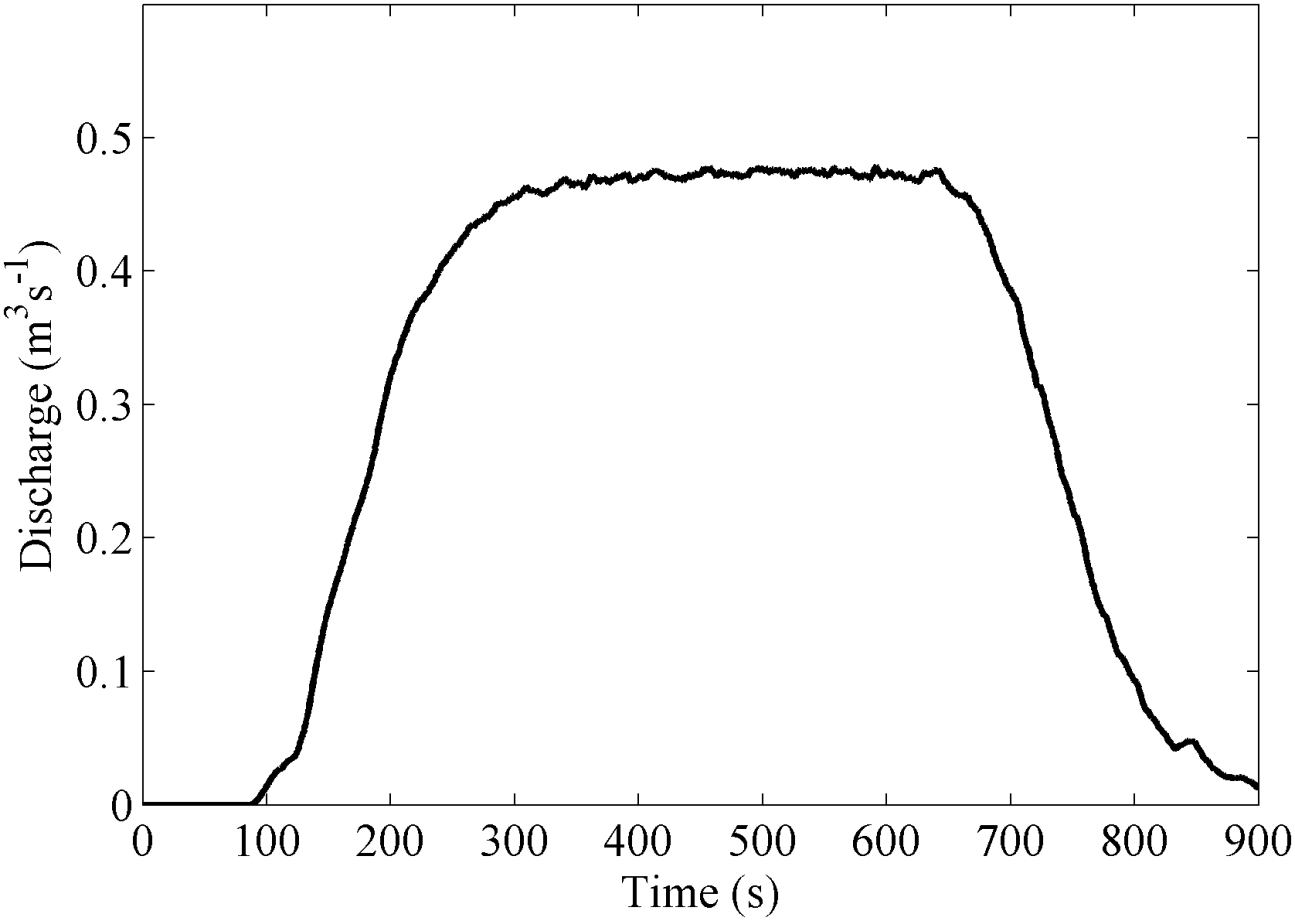
Water inflow at the upper boundary.

Figure 5 shows the time story of numerical water inflow at the upper boundary. The situation represents a short and intense rain event lasting about 15 minutes. Water inflow attains a constant value (around 0.49 m^3^s^−1^) at approximately 240 s, which lasts till 600 s when the inflow is slowly reduced.

The rain falling onto the simulated area is not considered since it is negligible compared to the water collected in the watershed. This can be estimated considering that the precipitation is on the order of 96 mm day^−1^ and the area of the computational domain is close to 6000 m^2^, the rain discharge inside the domain is 0.006 m^3^s^−1^, which is negligible when compared with the water inflow 0.5 m^3^s^−1^.

### Viscosity calibration for fluid-boundary interaction

As we mentioned before, the artificial viscosity corresponding to the interaction between fluid particles and a boundary that represents a terrain with vegetation should be calibrated. First *α_FF_* = 0.2 was imposed following previous research where similar values were considered ([11], [12]). Then, the model was calibrated to obtain *α_FB_* using an artificial channel that mimics the main features of the real terrain to be considered in the study (slope 0.17, width 2 m, length 73 m and water depth 0.04 m).

The Manning coefficients *n* is an empirical coefficient used on engineering representing the ease with which a flow descends a certain slope characterized by the kind of surface and the amount of debris existing in the channel of study. According to Manning equation
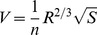
(9)the velocity (*V*) can be calculated in terms of the Manning coefficient (*n*), the hydraulic radius (*R*) and the slope of the channel (*S*).

**Figure 6:**
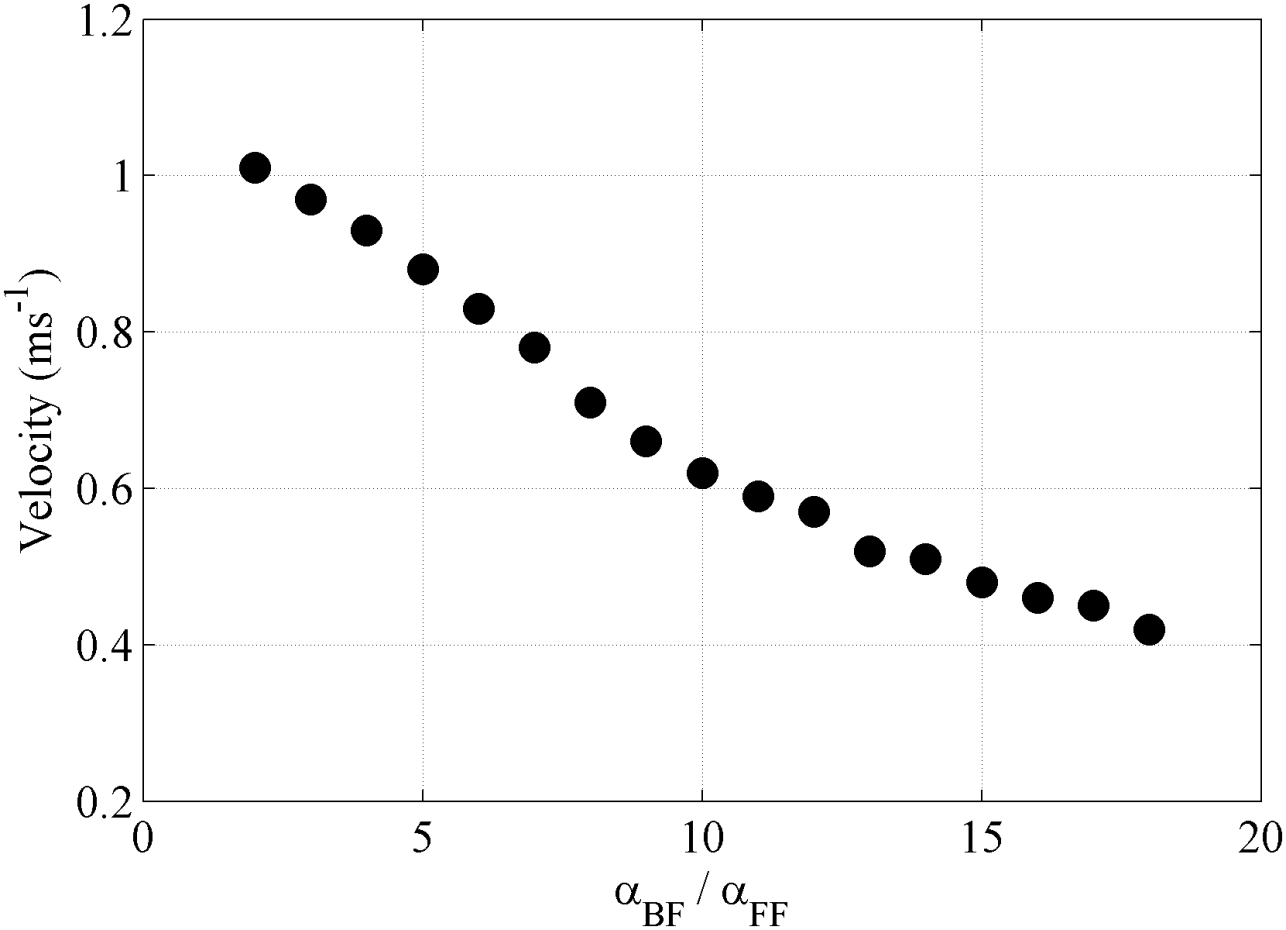
Flow velocity dependence on the ratio between *α_BF_* and *α_FF_*.

Taking into account the vegetation of the terrain under scope (area with heavy brush), the Manning coefficient is on the order of 0.075 (see Table 2), which results in a velocity of 0.46 ms^−1^ using Eq. (9). DualSPHysics was executed for different ratios between *α_FB_* and *α_FF_* (Figure 6) in order to obtain a velocity similar to the theoretical one, which was achieved for *α_FB_* = 16 *α_FF_*.

**Table 2:** Values of Manning coefficient according to the material of the surface.

Type of surface	Manning coefficient (*n*)
Glass	0.010±0.002
Concrete	0.012±0.002
Steel	0.014±0.003
Natural channel (low debris)	0.040±0.010
Area (low brush)	0.050±0.020
Area (high brush)	0.075±0.025

## Results

**Figure 7:**
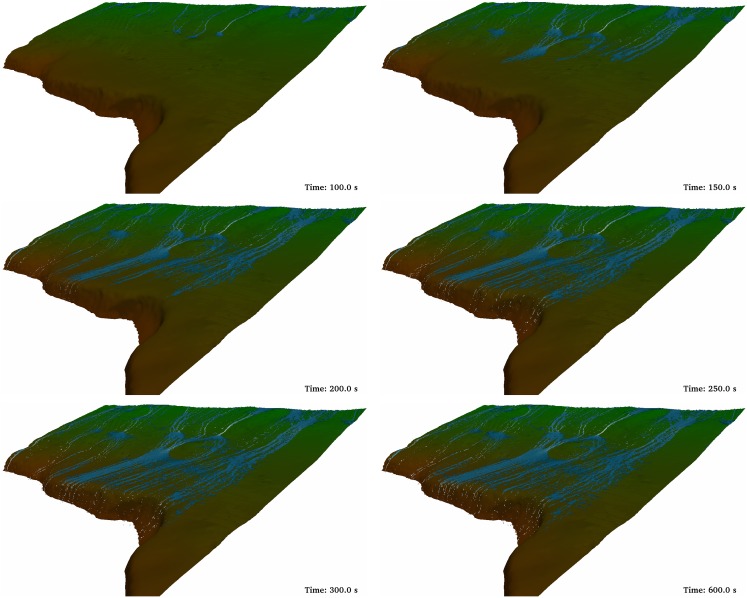
Different instants of the DualSPHysics simulation without ditch.

First, runoff was simulated without the ditch (Figure 7). In the first two panels water only affects the upper (Time = 100 s) and middle (Time = 150 s) parts of the computational domain. In the third panel (Time = 200 s) the water has already arrived at the lower part of the domain and falls into the road. From a computational point of view, the run in absence of ditch depicted in Figure 7 was carried out with 5,365,925 boundary particles. The number of fluid particles depended on the instant of the run due to the input/output conditions in the system. Thus, only 302,181 fluid particles were initially considered, but this number increases until 2,217,033 fluid particles at the moment of maximum flow. A runtime of 138 hours was necessary to run 900 seconds of physical time. All simulations were executed on a GPU Titan using double precision.

**Figure 8:**
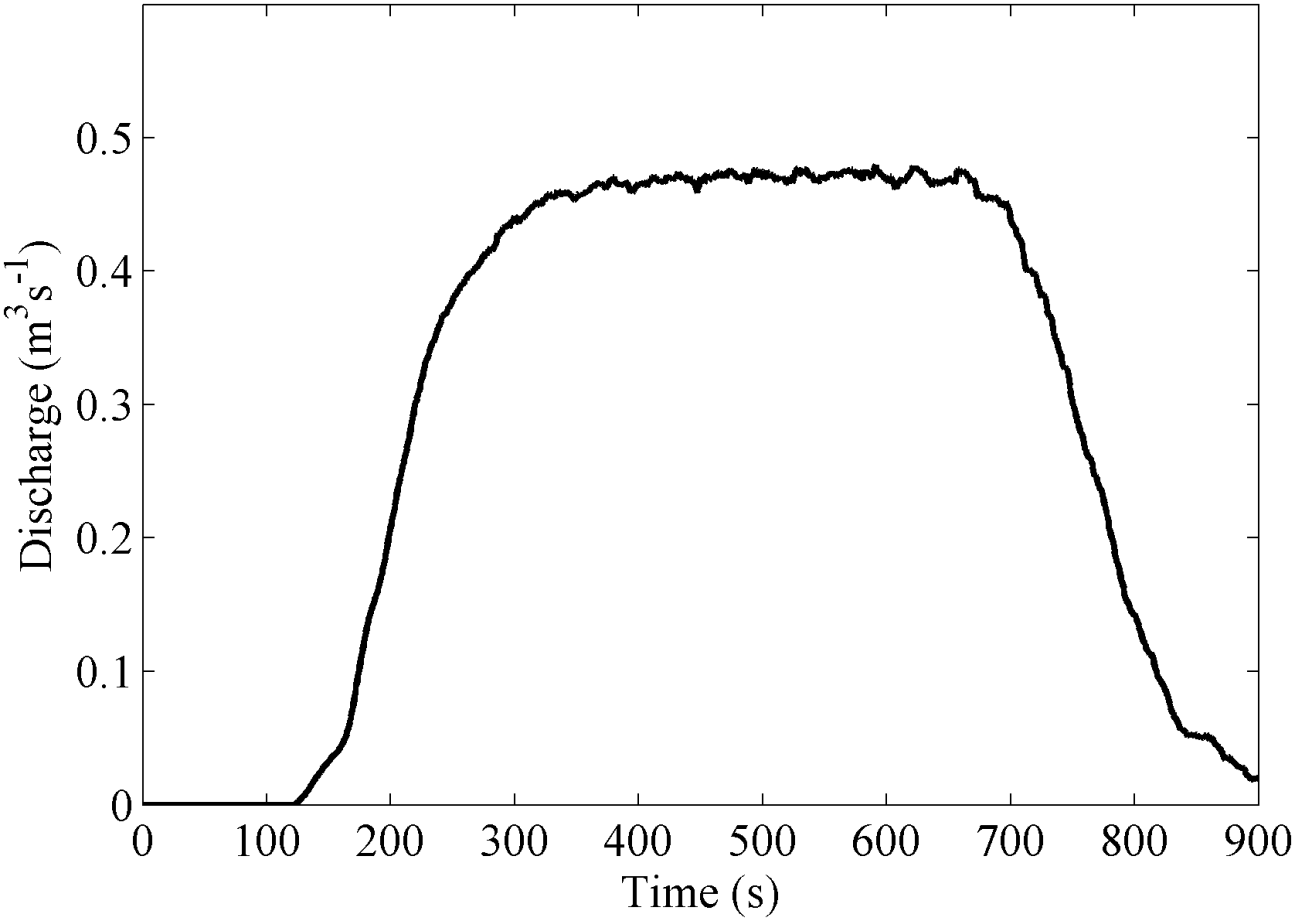
Water flow onto the road.

The amount of water arriving at the road can be seen in Figure 8. Basically, the plot is similar to the one showing the flow entering the computational domain (Figure 5). The maximum amount of water (around 0.46 m^3^s^−1^) is slightly smaller in Figure 8 because a small percentage of water (around 6%) leaves the computational domain through the open lateral boundaries. In addition, the signal in Figure 8 is delayed in around 50 seconds when compared with the signal in Figure 5. The flow takes around 50 seconds to reach the lower part of the region of interest since entering the computational domain, that is, travelling 73 m.

**Figure 9:**
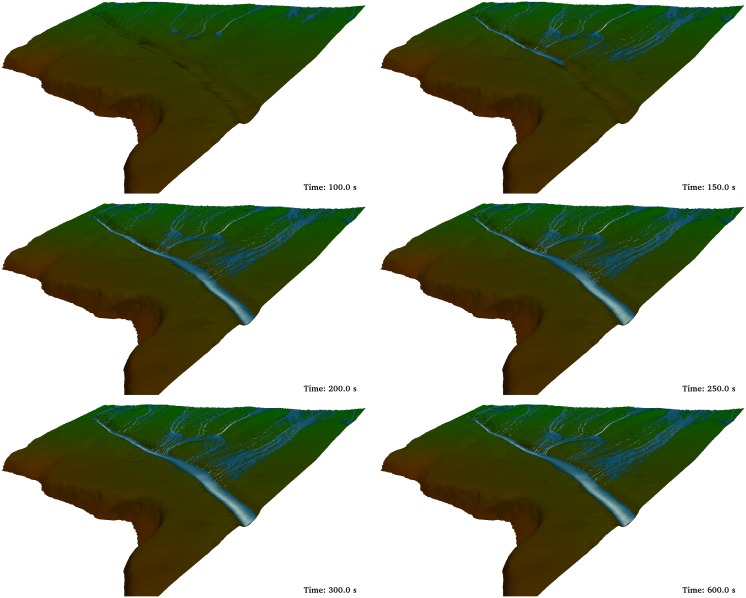
Different instants of the DualSPHysics simulation with a ditch 0.8 m deep. Additional detail about the simulation can be found at http://youtu.be/T1po4onk0v4.

Figure 9 shows the same discharge conditions as previously described for Figure 7. The only difference between both simulations is the presence of a ditch that mimics the main features of the real one shown in Figures 2 and 3; namely, 80 cm deep and 50 cm wide. The first frames are similar to the one in Figure 7. Nevertheless, the rest of the frames show how water is collected and drained by the ditch. Actually, there is not water discharge to the road under these conditions. The computational conditions are similar to the ones described above. The only difference is the maximum number of fluid particles, which is now 2,431,254, since the presence of the ditch increase the residence time of water inside the numerical domain. Consequently, the runtime also increased and it is now 150 hours.

**Figure 10:**
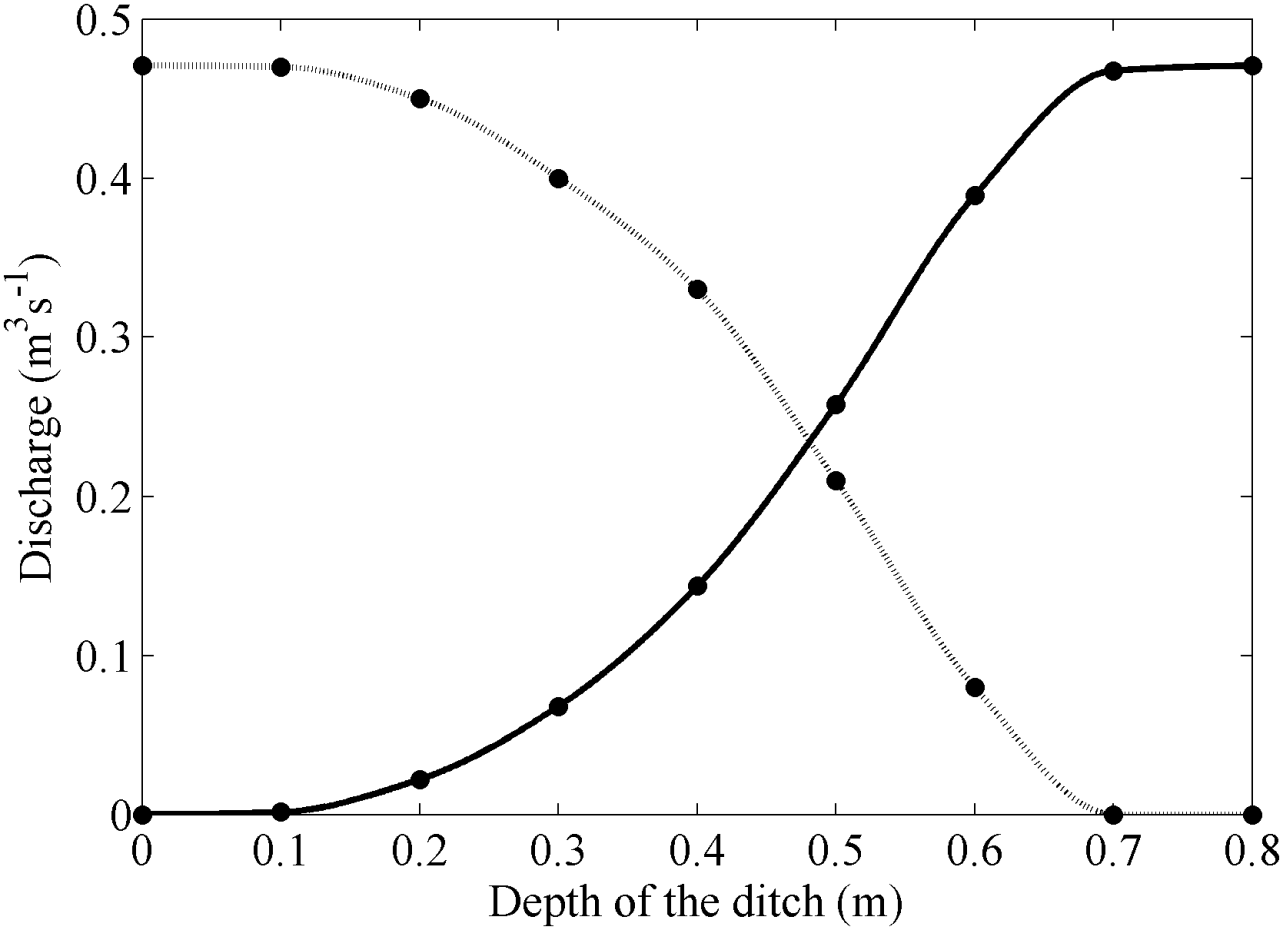
Discharge into the road (dashed line) and drainage of the ditch (solid line).

According to the plans of the builders, this ditch should be enough to drain water even under extreme runoffs. Nevertheless, ditches can become clogged with sediments or debris, especially after strong storm events. Here, the effect of sediment is also analysed, assuming that the maintenance of the ditch was enough to prevent clogs but not to prevent a slow sedimentation inside the ditch that reduced its depth. Figure 10 shows the water discharge into the road and the water drained by the ditch as a function of the depth of the ditch. The same water inflow (Figure 5) was considered in all simulations. The case with depth equal to zero corresponds to the absence of ditch analysed in Figures 7 and 8. Ditches with depths less than 0.2 m are shown to be inefficient under strong rain conditions and more than 90% of the discharge arrives at the road. The efficiency increases markedly for ditches deeper than 0.4 m, actually 50% of the discharge is drained by the ditch. Finally, a 0.7 m deep ditch is able to drain the 100% of the discharge under strong rain conditions.

## Conclusions

This study represents an approach to model the water runoff on complex geometries, with direct application in the field of civil engineering. The study merges two advanced technologies; namely, UAV photogrammetry and a fluid solver based on SPH technique.

UAV photogrammetry was used to obtain the geometry of the area. The point cloud data provided after photogrammetric restitution can be converted into points through a triangulation process and these points are used to create the initial setup of the SPH meshless method. DualSPHysics was applied to compute the water trajectories and its interaction with a complex terrain.

Fast and intense rain events were simulated using a water inflow that mimics extreme meteorological conditions in the zone that were calculated for a return period of 50 years. Thus, an inflow of about 0.5 m^3^s^−1^ was imposed through the upper boundary of the computational domain.

The effect of a ditch (0.8 m deep and 0.5 m wide) to prevent water flow into the road was analysed. This precautionary measure was observed to be effective to drain water under extreme conditions. Nevertheless, the efficiency of the ditch was observed to decrease when sedimentation reduces its depth, in such a way that the drainage was only on the order of 50% when the depth of the ditch decreased at about 0.45 m.

In summary, the combination of UAV measurements and DualSPHysics simulations has proven to be a suitable tool both to describe water floods on areas adjacent to the roads and to design measures to palliate those floods. The resulting tool can be applied to multiple civil engineering scenarios and give complementary information to the typical GIS solutions that exclusively use geometry to determine the water movement.
